# Multiplex STR amplification sensitivity in a silicon microchip

**DOI:** 10.1038/s41598-018-28229-9

**Published:** 2018-06-29

**Authors:** Senne Cornelis, Maarten Fauvart, Yannick Gansemans, Ann-Sophie Vander Plaetsen, Frederik Colle, Rodrigo S. Wiederkehr, Dieter Deforce, Tim Stakenborg, Filip Van Nieuwerburgh

**Affiliations:** 1 Laboratory of Pharmaceutical Biotechnology, Ghent University, 9000 Gent, Belgium; 2 Department of Life Sciences and Imaging, imec, 3001 Leuven, Belgium

## Abstract

The demand for solutions to perform forensic DNA profiling outside of centralized laboratories is increasing. We here demonstrate highly sensitive STR amplification using a silicon micro-PCR (µPCR) chip. Exploiting industry-standard semiconductor manufacturing processes, a device was fabricated that features a small form factor thanks to an integrated heating element covering three parallel micro-reactors with a reaction volume of 0.5 µl each. Diluted reference DNA samples (1 ng–31 pg) were amplified on the µPCR chip using the forensically validated AmpFISTR Identifier Plus kit, followed by conventional capillary electrophoresis. Complete STR profiles were generated with input DNA quantities down to 62 pg. Occasional allelic dropouts were observed from 31 pg downward. On-chip STR profiles were compared with those of identical samples amplified using a conventional thermal cycler for direct comparison of amplification sensitivity in a forensic setting. The observed sensitivity was in line with kit specifications for both µPCR and conventional PCR. Finally, a rapid amplification protocol was developed. Complete STR profiles could be generated in less than 17 minutes from as little as 125 pg template DNA. Together, our results are an important step towards the development of commercial, mass-produced, relatively cheap, handheld devices for on-site testing in forensic DNA analysis.

## Introduction

Currently, forensic DNA profiling is performed almost exclusively by multiplex polymerase chain reaction (PCR) amplification of short tandem repeat (STR) regions^1,2^. These repetitive sequences are highly polymorphic allowing for the generation of very distinctive profiles from minute amounts of DNA. The global consensus on using STRs allows for straightforward comparison of profiles in databases around the world^3,4^. To generate a forensic STR profile, a multiplex of several loci is amplified by PCR and subsequently analyzed using capillary electrophoresis (CE)^5^. Conventional PCR requires a thermal cycler with a large thermal mass, resulting in considerable thermal inertia. Consequently, the speed at which the instrument can be cooled or heated is limited. Furthermore, multiplex PCR amplification is an expensive, time-consuming and labor-intensive process requiring a dedicated laboratory setting with limited potential for on-site testing. Forensic laboratories around the world continually face the challenge of increased amounts of case work and the demand for timely and cost-effective analysis of the evidence^6^. In addition, there is an emerging demand for rapid DNA profiling that can be performed outside of centralized forensic laboratories, fueled by recent changes in legislation in the United States (Rapid DNA Act of 2017)^7^. Lab-on-a-chip technology could address some of the limitations hampering the conventional STR typing process^8–10^ and enable the development of Rapid DNA instruments. Its merit, with respect to conventional tools, is threefold: (1) Microfluidic platforms inherently require less reagents and sample, thus potentially decreasing the cost per analysis. (2) Silicon microchips possess favorable thermal properties allowing faster PCR cycling, thus shortening time to results^11,12^. (3) The potential of lab-on-chips for on-site analysis far exceeds that of conventional systems due to the smaller form factor and modest power consumption, permitting integration into portable devices. Several research groups from both academia and industry have been working towards fully integrated systems for STR profile generation, with a number of microfluidic systems described in the literature^13–17^. Previous efforts have focused chiefly on the analysis of so-called reference sample buccal swabs, obtained from known individuals and containing sufficient amounts of DNA for analysis. Consequently, amplification sensitivity, which is essential for achieving robust forensic DNA profiling of case samples, such as those obtained from a crime scene, was not studied in depth. In this work, the amplification sensitivity of a silicon micro-PCR (µPCR) chip for forensic PCR, produced using well-established semiconductor fabrication processes was directly compared to conventional amplification. Crucially, the use of industry-standard semiconductor manufacturing processes for the fabrication of prototype silicon µPCR chips guarantees cheap mass producibility, which is an important consideration for future commercial development of single-use disposable chips. The miniature chip holds three 0.5-µl reaction cavities for parallel testing and an integrated aluminum heater enabling rapid thermal cycling in a small form factor. Using the forensically validated AmpFISTR Identifier Plus Multiplex STR kit, the sensitivity of on-chip amplification was examined. A serial dilution of template material was amplified both on a conventional thermal cycler and on-chip. Detailed comparison of the resulting profiles demonstrated the capabilities of the µPCR chip to generate STR profiles that satisfy the strict quality requirements of forensic DNA analysis. Furthermore, a rapid amplification protocol was developed harnessing the full potential of on-chip amplification and allowing complete STR profiles to be generated in a fraction of the time normally required.

## Materials and Methods

### PCR chip design and fabrication

**Figure 1:**
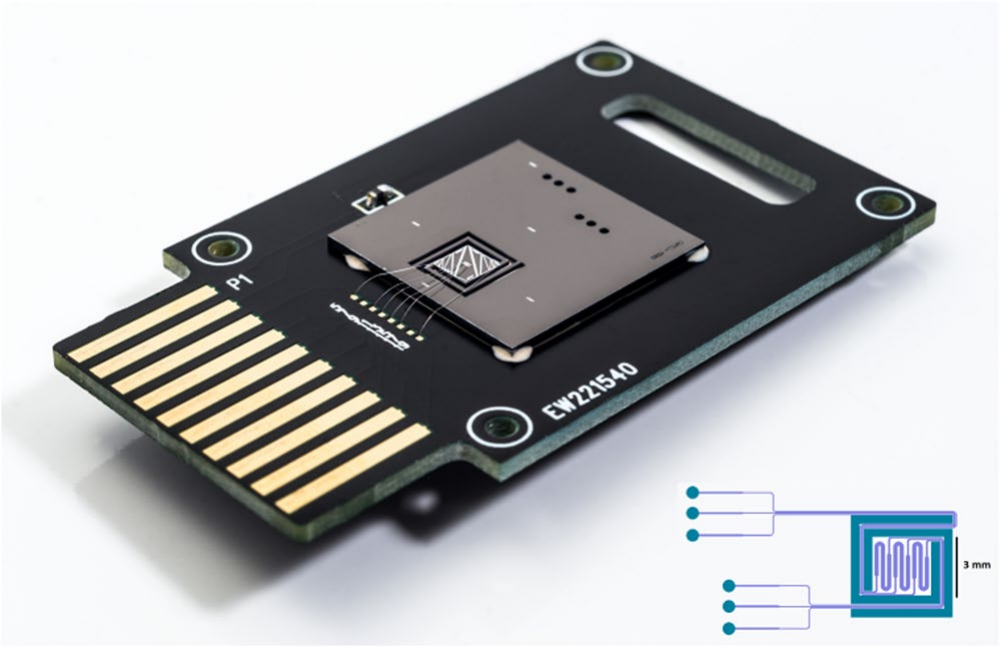
Top view showing the complete chip including integrated heater, air trenches surrounding the reaction cavity, three inlets and outlets and the PC. The integrated heater and resistance temperature detector are wire-bonded to the 10-pin connector. The insertion shows a schematic overview of the reaction chambers, access holes, channels and air trenches etched in silicon. Indicated in purple are the front side channels created by a front side etch, the blue layers represent the channels created by front and backside etching of the silicon substrate, thereby piercing through the silicon substrate. Depth of the front side etch and backside etch is 0.25 mm and 0.17 mm respectively.

The silicon µPCR chip used to perform amplification is depicted in Fig. 1. Each chip is composed of three PCR reactor cavities with individual inlet and outlet channels accessible from the back side. The microfluidic structures were etched in a 0.4 mm thick silicon wafer which was subsequently diced into 20 mm × 20 mm individual chips. The reaction cavities and associated microfluidic channels were created on the front side of the silicon substrate by dry etching 0.25 mm deep and subsequently sealed by anodic bonding of Pyrex. The air trenches for thermal isolation surrounding the reaction cavities, as well as the access ports for fluidics were created by a subsequent front and backside dry etching up to the Pyrex-silicon interface. The insertion in Fig. 1 shows a schematic representation of the chip structures etched in the silicon substrate. A detailed fabrication process was published previously by Majeed *et al*.^18^. Importantly, the entire fabrication process relies on industry-standard semiconductor manufacturing processes that are ideally suited for upscaling to mass production. The reactor cavity design was based on previous designs reported in literature^12,19,20^. With a total volume of 0.5 µl and a meandered shape lacking sharp curvature, the cavities are optimal to minimize air bubble formation during filling. Including the volume of the microfluidic channels, 0.8 µl is needed to fill the reactor cavity. To supply heat to the micro-reactors we used a µPCR chip equipped with an integrated 300 nm thick aluminum Joule heater recently developed by Barman *et al*.^21^. This micro-heater design was optimized to reduce non-uniform heating with a spread of +/−0.5 °C at 95 °C, with the largest differences in the inlet and outlet regions. The specific design of the heater and surrounding air-trenches result in localized heating and decreased heat dissipation during periods of rapid heating. Thus, in combination with the inherently small reaction volume and thermal conductivity properties of silicon (130 W/m^2^ K), a highly efficient heat transfer is achieved, resulting in a maximum heating rate of 6.6 °C/s. Furthermore, an active cooling system was deemed unnecessary. Solely relying on natural convection, a cooling rate identical to the heating rate (6.6 °C/s) could be achieved. The lack of an active cooling system was intentional to keep the design and fabrication simple. Temperature monitoring was accomplished by a calibrated resistance temperature detector (RTD) placed alongside the heater. The chip was bound to a printed circuit board (PCB) using a thermally non-conductive epoxy. The heater and temperature sensor were connected to the PCB pins using 25 µm aluminum wires. A standard 10-pin socket connected the PCB with a temperature controller. The reagents were loaded into the chip using a micropipette placed at the inlet holes.

### Conventional (off-chip) PCR

Conventional (off-chip) amplification was carried out using 0.2 ml thin-walled reaction strips (Westburg, The Netherlands) and the SimpliAmp Thermal Cycler (Applied Biosystems, USA). The reaction volume was 25 µl as prescribed by the AmpFiSTR® Identifiler Plus kit specifications. This conventional PCR amplification method is referred to in this report as “off-chip” amplification.

### Off- and on-chip PCR with identical template amounts

The AmpFISTR ® Identifier Plus PCR Amplification Kit (Applied Biosystems, USA) was used to amplify the amelogenin locus and the following tetrameric STR loci: CSF1PO, D2S1338, D3S1358, D5S818, D7S820, D8S1179, D13S317, D16S539, D18S51, D19S433, D21S11, FGA, TH01, TPOX, vWA. Female Human Forensic Control DNA 9947A in TE buffer (Origene, USA) was used as reference sample and serially diluted ranging from 1 ng to 31 pg. A no template control (NTC) experiment containing water instead of DNA template was added to rule out any non-specific amplification. All PCR reactions were conducted according to the manufacturer’s recommendations using a 29-cycle PCR protocol for added sensitivity when amplifying DNA input amounts lower than 125 pg. The temperature profile consisted of an initial denaturation at 95 °C for 11 min, followed by thermal cycling including denaturation at 94 °C for 20 s, primer annealing and extension at 59 °C for 3 min and a final elongation step of 10 min at 60 °C. The heating and cooling ramp rates were restricted to 5 °C/s, which is well within the limitations of the integrated heating system. Identical concentrations of the AmpFISTR Identifiler Plus Master Mix and Primer Set were used for off- and on-chip amplification. No specific optimization of the kit protocol, other than reducing the volumes (50-fold reduction), was performed to allow on-chip amplification. The absolute amount of DNA template input was maintained in conventional and on-chip amplification (1 ng–31 pg), implying a 50-fold increase of the DNA concentration in the on-chip reaction mixture (cfr. 50-fold reduction in reaction volume). Replicate amplifications (n = 3) were performed on different chips and in different reaction cavities, to measure the chip-to-chip and run-to-run variation.

### Off- and on-chip PCR with identical template concentrations

Additional experiments were conducted comparing the conventional and on-chip PCR amplification using samples of identical concentration. Samples containing 0.1 ng/µl, 0.05 ng/µl and 0.025 ng/µl template were amplified. Following the manufacturer’s instructions, the conventional PCR reaction has a total reaction volume of 25 µl, of which 10 µl should be the DNA sample volume. Using abovementioned sample concentrations, this results in 1 ng, 500 pg and 250 pg input DNA respectively. The on-chip reaction volume contains only 0.2 µl DNA sample, corresponding to 20 pg, 10 pg and 5 pg input DNA respectively.

### Rapid on-chip PCR

To harness the full potential of the favorable thermal properties of silicon microchips, a rapid on-chip PCR amplification protocol was designed. This customized protocol includes an optimized master mix and temperature profile. The master mix was developed based on the SpeedSTAR HS DNA Polymerase kit (Takara, USA) following the manufacturer’s instructions, with some optimization. Each reaction consisted of 0.12 U SpeedSTAR HS DNA Polymerase, 1X Fast Buffer I, 250 µM dNTPs. Identifiler Plus primer concentrations and input DNA quantities were identical to those used for the comparison between off- and on-chip PCR (section 2.2) at conventional speed. The Fast SpeedSTAR HS DNA Polymerase can incorporate 1 kb in less than 10 seconds, allowing a considerable reduction of elongation times. The temperature profile consisted of an initial denaturation at 95 °C for 1 min, followed by thermal cycling including denaturation at 94 °C for 5 s and primer annealing and extension at 62 °C for 12 s, and a final elongation step of 1 min 30 s at 60 °C. The primer annealing and extension temperature (62 °C) was optimized by off-chip optimization experiments. This temperature allowed the SpeedSTAR polymerase to properly elongate while the Identifiler plus primers could still readily bind to the target sequences. Similar optimization experiments conducted by Foster and Laurin yielded a comparable (61 °C) annealing and extension temperature^22^. The total amplification time was further reduced by increasing the heating and cooling ramp rates to 6.6 °C/s, resulting in an amplification time of less than 17 min. Rapid amplification was conducted for samples ranging from 1 ng to 32 pg. Again, all experiments were performed in triplicates using different chips and different reaction cavities to measure the chip-to-chip and run-to-run variation.

### CE analysis

STR profiles were generated using high-resolution capillary electrophoresis. The on-chip PCR products were manually recovered from the reaction cavities. All samples were subsequently analyzed on an Applied Biosystems 3130xl Genetic analyzer using 36-cm capillaries containing POP-4™ matrix (Applied Biosystems) at 60 °C as per the manufacturer’s instructions. One alteration to the standard protocol was made, the recommended sample input of 1 µl was lowered to match the volume retrieved from the chip reaction cavity (0.5 µl). All samples were added to a Hi-Di™ formamide (Applied Biosystems) - GeneScan™ 500 LIZ Size Standard (Applied Biosystems) mixture. Samples were heated at 95 °C for 3 min, snap-cooled and analyzed using a run module corresponding to a 22 s sample injection time at 3 kV and 2200 s separation time at 15 kV. Electropherograms were analyzed using GeneMapper® ID Analysis software v3.2 (Applied Biosystems). PCR sensitivity was assessed based on the presence of the 26 possible alleles in the STR profile. Alleles with peaks heights greater than 50 RFU were considered present while the other alleles were considered absent. The allelic balance of heterozygous loci, calculated as the ratio of the peak heights of both alleles was also assessed. As the genotypes of all samples used in this study were known, no homozygous/heterozygous threshold was used.

## Results

### Off- and on-chip PCR with identical template quantities

**Figure 2:**
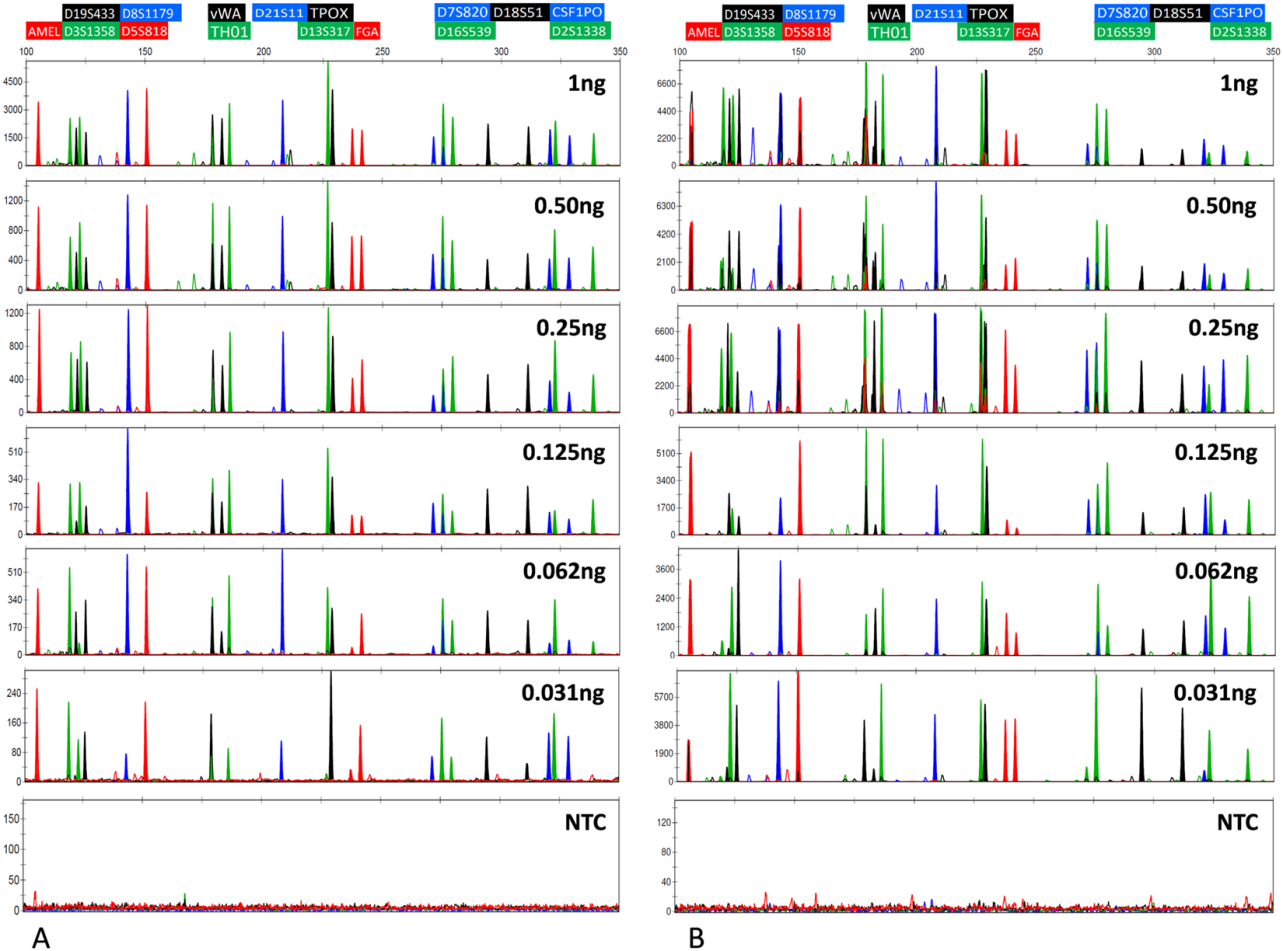
CE profiles generated after (**A**) Off-chip amplification and (**B**) On-chip amplification of a serial dilution of input material (1 ng–31 pg) and a no template control (NTC). All experiments were conducted in triplicates (n = 3).

To demonstrate the µPCR chip’s multiplex amplification functionality, a sensitivity study was performed comparing forensic STR profiles generated both by on-chip and off-chip amplification. First, STR profiles were generated using off-chip amplification after serial dilution of the 9947A reference template DNA (Fig. 2A). Complete STR profiles could be obtained throughout the entire dilution series. Unbalanced amplification of some alleles was observed, more specifically at lower DNA input quantities (≤62 pg). Some samples with 31 pg DNA input showed partial profiles with several allelic dropouts. The observed sensitivity was in line with the kit specifications which states that for the 29-cycle amplification protocol full profiles (26 alleles) should consistently be obtained at 62 pg and occasional partial profiles may be observed at 31 pg.

**Figure 3:**
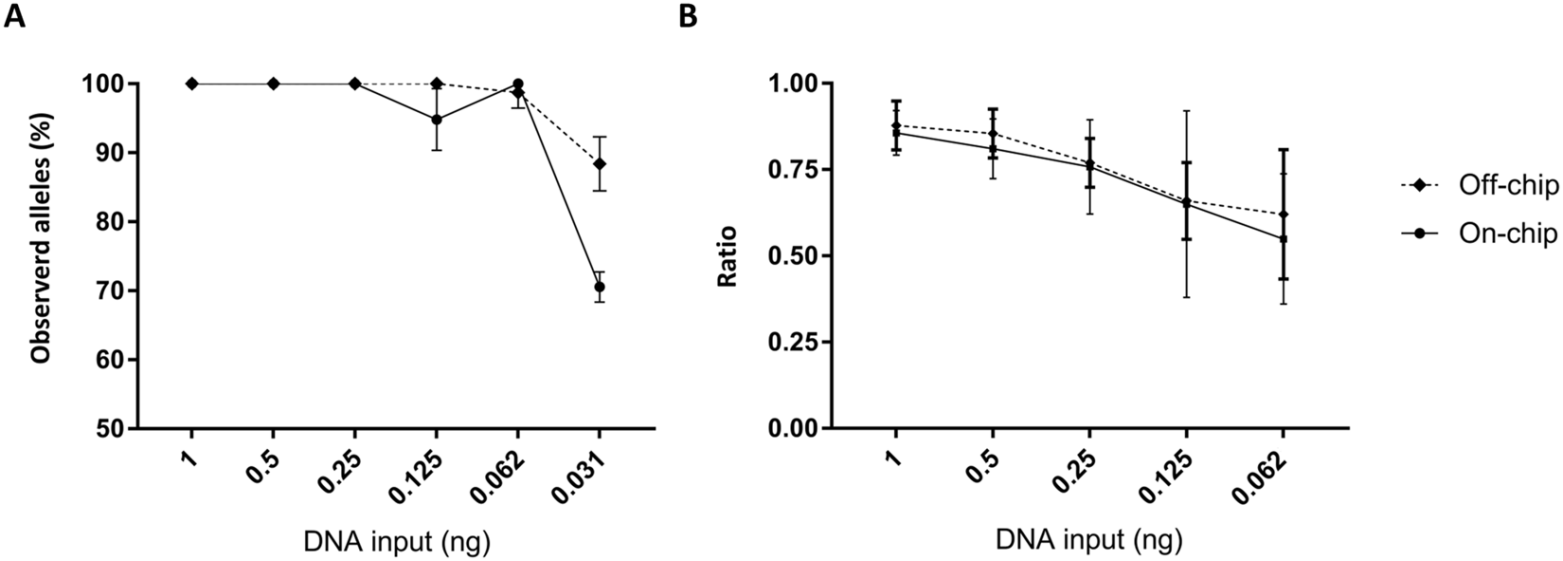
(**A**) Percentage of observed alleles throughout the dilution series for both Off-chip (♦) and On-chip (●) amplifications. (**B**) Peak height ratio of all heterozygous loci throughout the dilution series for both Off-chip (♦) and On-chip (●) amplifications. All amplifications were conducted in triplicates (n = 3).

Next, on-chip STR profiling was compared to conventional off-chip analysis. The small PCR reactor cavities, with a volume of just 0.5 µl, result in a decreased consumption of reagents and forensic sample, leading to cost savings. A serial dilution was used to match input quantities of the off-chip amplification experiments. Resulting profiles (Fig. 2B) were compared to their off-chip counterparts (Fig. 2A). Figure 3A gives a representation of the number of dropouts by showing the percentage of observed alleles. The extent of dropouts is comparable for on-chip and off-chip amplification: With only one exception, dropouts were only observed when samples with input quantities lower than 62 pg were amplified. Compared to off-chip amplification, on-chip amplification of these low input quantities consistently resulted in a slightly higher number of dropouts. The on-chip STR profiles originating from samples with high amounts of input material (1 ng, 500 pg and 250 pg, 125 pg) produced complete profiles that can be readily interpreted. However, several peaks caused by spectral bleed-through could be observed. The lower input range (62 pg–31 pg) seemed less affected by this phenomenon. The heterozygous allelic balance for both on-chip and off-chip amplification is represented in Fig. 3B as the average (n = 3) peak height ratio (RFU peak 1/RFU peak 2). A comparable peak height ratio was observed for both methods of amplification. A strong correlation exists between the allelic peak height ratio and the amount of input material used during the amplification. High input samples consistently resulted a heterozygous ratio above 0.75 (i.e. low allelic imbalance), whereas a ratio between 0.75 and 0.5 (i.e. more allelic imbalance) was observed for the samples containing less than 250 pg. Even though no average allelic peak height ratio lower than 0.5 was observed, some low input samples did display considerable sample-to-sample and locus-to-locus fluctuations of the peak height ratio. Locus-specific allelic balance ratios below 0.5 as well as several allelic dropouts were frequently observed in samples containing 32 pg DNA. Together, these results demonstrate equivalent efficiency for both on- and off-chip STR profiling.

### Off- and on-chip PCR with identical template concentrations

**Figure 4:**
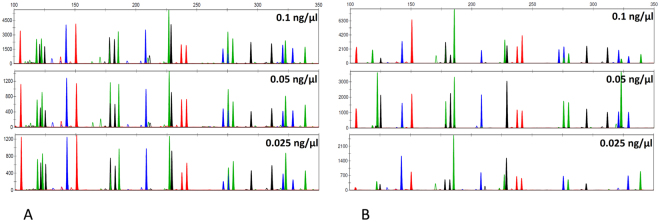
Comparison of CE profiles generated Off-chip (**A**) and On-chip (**B**) using identical input concentrations (0.1 ng/µl, 0.05 ng/µl, 0.025 ng/µl). All experiments were conducted in triplicates (n = 3).

On-chip and off-chip amplification of samples containing template DNA concentrations of 0.1 ng/µl, 0.05 ng/µl and 0.025 ng/µl were compared (Fig. 4). Amplifying these samples off-chip, following the kit’s instruction (10 µl DNA sample per reaction), correspond to amplifying 1 ng, 500 pg and 250 pg input DNA respectively (Fig. 4A). Amplifying the same samples on-chip corresponds to template DNA quantities of 20 pg, 10 pg and 5 pg respectively, due to the 50-fold reduction in reaction volume. The resulting on-chip profiles show severe allelic imbalance and dropout, only producing an occasional full profile for a DNA input concentration of 0.1 ng/µl (Fig. 4B).

### Rapid on-chip PCR

**Figure 5:**
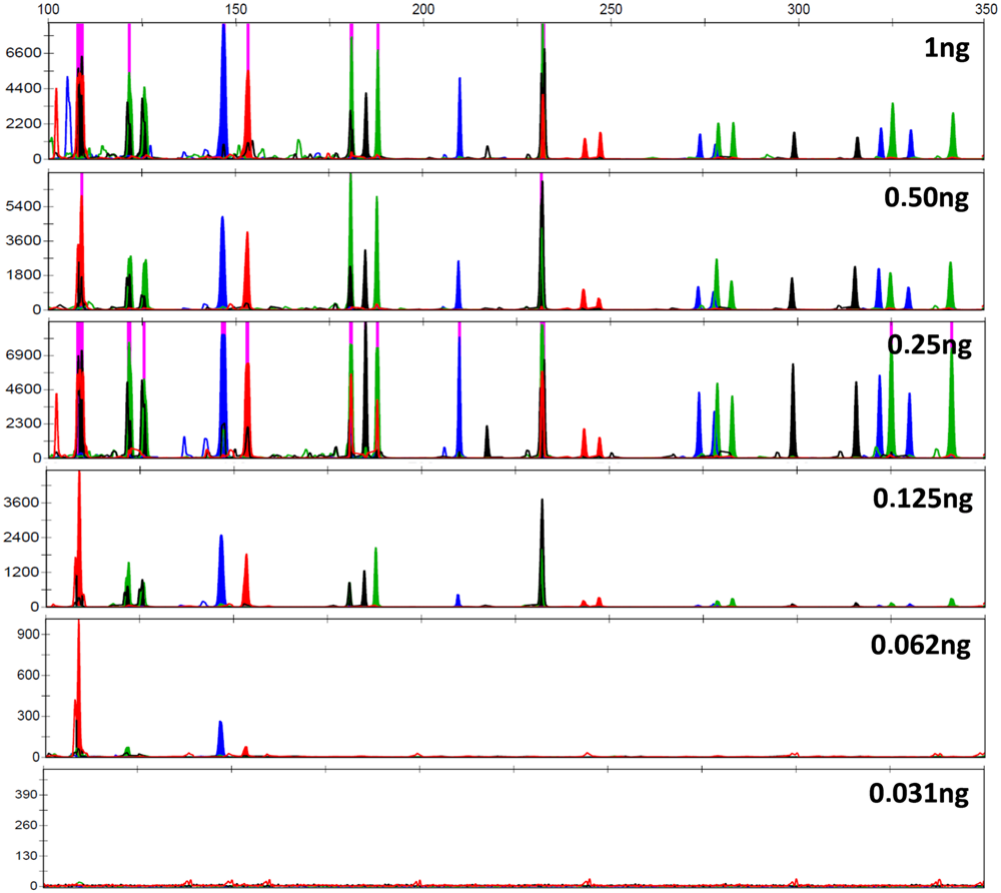
CE profiles generated after rapid on-chip PCR amplification of a serial dilution of input material (1 ng–31 pg). All experiments were conducted in triplicates (n = 3).

Rapid amplification experiments were conducted using a combination of the Identifiler Plus primer set and the SpeedSTAR HS polymerase and master mix. After optimization of the temperature profile, PCR amplification could be performed in less than 17 minutes. A serial dilution (1 ng, 500 pg and 250 pg, 125 pg, 62 pg, 31 pg) was amplified and subsequently analyzed by CE. Representative CE profiles resulting from the rapid PCR amplification protocol are shown in Fig. 5. Supplementary Fig. 1A gives a representation of the number of dropouts by showing the percentage of observed alleles for each input quantity. Full profiles could consistently be observed with input quantities of 250 pg and higher. Amplifications conducted with samples containing 125 pg template DNA generally produced full profiles, however some sporadic dropouts were observed. The observed dropouts predominantly occurred with the CSF1PO, D2S1338, D18S51, D7S820 and D16S539 loci. All of these loci are longer than 250 bp. Samples containing 62 pg and 31 pg produced no useable profiles. Some sporadic amplification of the shortest loci, such as the amelogenin locus could be observed. The heterozygous allelic balance is represented in Supplementary Fig. 1B as the average (n = 3) peak height ratio (RFU peak 1/RFU peak 2). Consistent with the results obtained with off-chip and “slow” on-chip amplification, 1 ng input samples resulted a heterozygous ratio above 0.75 whereas a ratio between 0.75 and 0.5 (i.e. more allelic imbalance) was observed for the samples containing 500 pg to 125 pg. No reliable peak height ratio could be calculated for the 62 pg and 32 pg samples as the dropout rate was too high.

## Discussion

Significant efforts have been made both in academia and industry to produce fully integrated systems for STR profile generation^14–17,23–25^. Examples include the plastic cartridge that incorporates purification, amplification and glass-based capillary electrophoresis reported by Hopwood *et al*.^26^, and the partially integrated chip, fabricated in glass, described by Bienvenue *et al*.^27^. An early silicon-based system has been reported by Schmalzing *et al*.^28^. More recently, Petralia *et al*.^29^ reported a silicon-based total analysis system. Commercial examples of automated Rapid DNA analysis systems include RapidHIT ID (IntegenX) and DNAscan/ANDE (NetBio). In line with guidelines from the FBI, Rapid DNA analysis efforts have focused on reference sample buccal swabs obtained from known individuals and containing sufficient amounts of DNA for analysis. For this reason, amplification sensitivity of integrated STR profiling solutions has largely been ignored. For robust forensic DNA profiling of case samples, such as those obtained from a crime scene, however, sensitivity is of critical importance. In this study, we therefore investigated the potential of a silicon µPCR chip for forensic DNA profiling and compared its amplification sensitivity with a conventional thermal cycler. The advantages of the proposed silicon chip-based solution to be incorporated into a Rapid DNA instrument are threefold: (i) integrated heaters and sub-µl reaction volumes allow for a small form factor; (ii) small PCR reactors etched into thermally conductive silicon, surrounded by thermal insulation trenches, enable rapid thermal cycling with limited power consumption; (iii) chip fabrication using industry-standard semiconductor manufacturing processes enable highly reproducible and relatively cheap mass production. Together, these factors enable the integration of our µPCR chip in truly portable, commercial devices for on-site STR profiling.

To assess on-chip STR amplification sensitivity, a forensic STR multiplex PCR kit was used to amplify varying amounts of the 9947A reference DNA sample, either in a common benchtop thermocycler (off-chip) or on the herein presented µPCR chip (on-chip). No specific optimization to the Identifiler Plus protocol, except for reducing the reaction volume, was necessary to obtain on-chip profiles that were in almost perfect concordance with their off-chip counterparts, demonstrating equivalent sensitivity for both on- and off-chip STR profiling. The extent of dropouts is comparable for on-chip and off-chip amplification. With one exception, dropouts were only observed when samples with input quantities lower than 62 pg were amplified. On-chip amplification of these low input amounts consistently resulted in a slightly higher dropout rate. A comparable heterozygous peak height ratio was observed for both amplification methods. Profiles resulting from the higher amounts of input DNA, suffer from peaks that are the result of spectral bleed-through of other peaks and suffer from peak splitting. Bleed-through is not uncommon with the analysis of highly concentrated samples. Peak splitting can also be directly linked to high input amounts. As more PCR product is present, the polymerase needs more time perform A-tailing resulting in an incomplete +A nucleotide addition to the final PCR product and hence split peaks can be observed^30,31^. Based on the results described here an optimal sample quantity range of 250 pg and 125 pg DNA is proposed for on-chip amplification. To achieve such sample quantities in the 0.5 µl reaction cavity, sample concentrations of 1.25 ng/µl and 0.625 ng/µl are respectively required in our setting. Comparable concentrations are encountered in crime scene cases. However, it is not improbable that in some cases (i.e. trace samples) these concentrations cannot be obtained. To further compare this, samples with a concentration of 0.1 ng/µl, 0.05 ng/µl and 0.025 ng/µl were amplified both on-chip and off-chip rather than comparing the amplification of identical absolute template quantities. On-chip amplification of the 0.1 ng/µl samples resulted in occasional full profiles and partial profiles were observed when amplifying the 0.025 ng/µl samples. Despite allelic imbalance and dropouts on-chip amplification using extremely low input amounts was demonstrated. While insufficient for typing, these partial profiles may provide sufficient information to exclude potential suspects, which is useful in an early identification. Several approaches to increase the sample concentration have been described in literature, including Silica-based kits (QIAamp®, Qiagen, USA), centrifugal filter devices (Microcon®, Merck Millipore, Germany) and vacuum concentrators (Speedvac, Thermo Fisher, USA)^32,33^. However, these added sample preparation steps do introduce additional hands-on laboratory time and require trained staff. Consequently, we envisage that the on-chip amplification described here would predominantly be used for the amplification of reference samples. These reference samples are always collected using standard methods (usually buccal swabs) making them significantly easier to analyze. Moreover, buccal swabs provide an excellent source of DNA resulting in a relatively high DNA concentration, well suited for on-chip amplification^34^. On-chip amplification after single cell isolation could be a potential field of interest^35^. Ballantyne *et al*.^36^ already demonstrated the potential of STR typing after whole-genome amplification, supporting the feasibility of this approach in forensic DNA analysis.

The favorable thermal properties of silicon for temperature cycling were exploited to speed up on-chip PCR amplification. With a total amplification time of less than 17 minutes, a 7-fold reduction was achieved compared to the conventional off-chip amplification time. Even though singleplex amplifications have already been reported to be conducted in under 10 minutes^37^, STR multiplex amplification remains more challenging in terms of speed as many fragments need to be amplified in a multiplex PCR^38^. Reducing the amplification time of the 16-plex forensic Identifiler plus assay did come at a cost, more specifically a reduced sensitivity. Complete STR profiles could only be generated with samples containing a least 125 pg to 250 pg template DNA. Moreover, at 125 pg a correlation between the amplicon size and PCR efficiency became apparent. A ‘ski-slope’ pattern was observed, with the smaller amplicons having a stronger signal compared to the signal intensities of larger amplicons such as CSF1PO, D2S1338 and D18S51. This ‘ski-slope’ electropherogram is a strong indication that the rapid PCR efficiency is dependent on amplicon size^39,40^. Not surprisingly, in one of the 125 pg containing triplicate samples several dropouts were observed, all of which were longer than 250 bp. The results presented here are in accordance with the comparative study presented by Butts and Vallone^41^ in which six different benchtop thermal cycling platforms were tested for their potential to perform rapid PCR amplification in a forensic setting. Using an identical primer set, the sensitivity presented here, using the on-chip amplification, is slightly better then what was reported for these six thermal cyclers. A potential explanation for the increased sensitivity when performing rapid amplification on-chip compared to traditional benchtop thermal cyclers are the differences in thermal mass and inertia. Conventional thermal cyclers rarely achieve fast cycling conditions due to their large thermal mass. Even if these rapid ramp rates are achieved, temperature control is typically less accurate. Rapid benchtop thermal cyclers typically overshoot the set temperature. In contrast, the sample is presumed to be heated to the correct temperature^42,43^. Because of the small thermal inertia of the chip being used in this paper, no such overheating is necessary.

## Conclusion

This work compared the sensitivity of the forensic AmpFlSTR Identifiler Plus kit using a conventional thermal cycler and an integrated µPCR chip. The potential of the integrated µPCR chip was demonstrated without the need for any specific optimization to the multiplex PCR kit protocol. A nearly identical PCR sensitivity between the standard off-chip and the integrated µPCR chip amplification was observed. Furthermore, the potential for rapid PCR amplification in a forensic setting was investigated. A 7-fold amplification time reduction compared to conventional instrumentation was achieved, while still obtaining an acceptable sensitivity. Taken together, our results show that multiplex PCR amplification, an essential part of forensic DNA analysis, can readily be performed in a silicon µPCR chip with comparable sensitivity even at an increased speed. This is an important step towards the development of a handheld forensic analysis tool for on-site DNA testing.

## Electronic supplementary material

Supplementary Figure 1
