# Supplementary material for: Multiplex STR amplification sensitivity in a silicon microchip
Source: Sci Rep. 2018 Jun 29;8:9853. doi: 10.1038/s41598-018-28229-9 (PMC6026139; doi:10.1038/s41598-018-28229-9)
Supplement: Supplementary file 1 — Supplementary Figure 1 [file 41598_2018_28229_MOESM1_ESM.pdf]

SUPPLEMENTARY INFORMATION

Multiplex STR amplification sensitivity in a silicon microchip

AUTHORS

Senne Cornelis[1,2], Maarten Fauvart[2], Yannick Gansemans[1], Ann-Sophie Vander Plaetsen[1], Frederik Colle[2], Rodrigo S Wiederkehr[2], Dieter Deforce[1,#,*] , Tim Stakenborg[2,#], Filip Van Nieuwerburgh[1,#]

[1] Laboratory of Pharmaceutical Biotechnology, Ghent University, 9000 Gent, Belgium

[2] Department of Life Sciences and Imaging, imec, 3001 Leuven, Belgium.

# Contributed equally

* Corresponding author at: Laboratory of Pharmaceutical Biotechnology, Faculty of Pharmaceutical Sciences, Ghent University, Ottergemsesteenweg 460, 9000 Gent, Belgium.

Tel: +32 (0)9 264 80 48

Email addresses:
Senne Cornelis: Senne.Cornelis@UGent.be,
Maarten Fauvart: Maarten.Fauvart@imec.be,
Yannick Gansemans: Yannick.Gansemans@UGent.be,
Ann-Sophie Vander Plaetsen: AnnSophie.VanderPlaetsen@UGent.be,
Frederik Colle : Frederik.Colle@imec.be,
Rodrigo Sergio Wiederkehr: Rodrigo.Sergio.Wiederkehr@imec.be,
Dieter Deforce: Dieter.Deforce@UGent.be,
Tim Stakenborg: Tim.Stakenborg@imec.be,
Filip Van Nieuwerburgh: Filip.VanNieuwerburgh@UGent.be

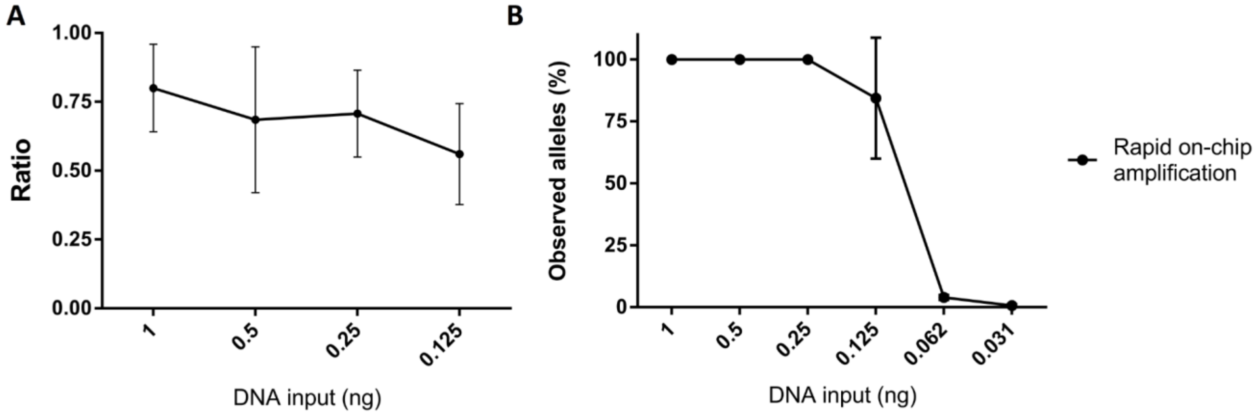

Supplementary Fig. 1: **A)** Percentage of observed alleles throughout the dilution series subsequent to rapid on-chip amplification. **B)** Peak height ratio of all heterozygous loci throughout the dilution series subsequent to rapid on-chip amplification. No reliable peak height ratio could be calculated for the 62 pg and 32 pg samples as the dropout rate was too high. All amplifications were conducted in triplicates (n=3).
